# Preoperative synovial fluid culture poorly predicts the pathogen causing periprosthetic joint infection

**DOI:** 10.1007/s15010-020-01540-2

**Published:** 2020-11-03

**Authors:** Philipp Schulz, Constantin E. Dlaska, Carsten Perka, Andrej Trampuz, Nora Renz

**Affiliations:** 1 Charité - Universitätsmedizin Berlin, corporate member of Freie Universität Berlin, Humboldt-Universität zu Berlin, and Berlin Institute of Health, Center for Musculoskeletal Surgery (CMSC), Charitéplatz 1, 10117 Berlin, Germany; 2 Orthopaedic Research Institute of Queensland, 7 Turner Street, Pimlico, Townsville, QLD 4812 Australia

**Keywords:** Synovial fluid, Pathogen, Detection, Periprosthetic joint infection, Preoperative arthrocentesis

## Abstract

**Purpose:**

We investigated the value of preoperative pathogen detection and evaluated its concordance with intraoperative cultures in patients with culture-positive periprosthetic joint infection (PJI).

**Methods:**

Culture-positive PJI episodes with available preoperative (synovial fluid) and intraoperative cultures (periprosthetic tissue, synovial or sonication fluid) were analyzed. The pathogen detection rate in preoperative and intraoperative cultures was compared using Fisher’s exact test and their concordance was calculated.

**Results:**

Among 167 included PJI episodes, 150 were monomicrobial with coagulase-negative staphylococci (*n* = 55, 37%), *S. aureus* (*n* = 34, 23%), and streptococci (*n* = 21, 14%) being the most common pathogens. Seventeen episodes (10%) were polymicrobial infections. The pathogen(s) grew in preoperative culture in 110 and in intraoperative cultures in 153 episodes (66% vs. 92%, *p* < 0.001). The pathogen detection rate was lower in preoperative compared to intraoperative cultures for low-virulent pathogens (40% vs. 94%, *p* < 0.001), polymicrobial infections (59% vs. 100%, *p* = 0.007), and in delayed and late PJI (63% vs. 94%, and 66% vs. 91%, respectively, *p* < 0.001). Full concordance of preoperative and intraoperative cultures was found in 87 episodes (52%). The pathogen was detected solely preoperatively in 14 episodes (8%) and solely intraoperatively in 57 cases (34%); an additional pathogen was found in 3 episodes (2%) preoperatively and in 6 episodes (4%) intraoperatively.

**Conclusion:**

The concordance of preoperative and intraoperative cultures was poor (52%). The sole or an additional pathogen was found exclusively in intraoperative cultures in 38% of PJI episodes, hence preoperative synovial fluid cultures are considered unreliable for pathogen detection in PJI.

## Introduction

Despite considerable progress in diagnostic and therapeutic management, periprosthetic joint infection (PJI) remains an ongoing challenge that requires a multidisciplinary approach. The lack of a single diagnostic test for PJI entails a multimodal work-up procedure including clinical, cytological, histopathological, and microbiological investigations [1, 2].

Pre- and intraoperative microbiological cultures are considered the gold standard for the diagnosis of PJI [3, 4]. The identification of the causative pathogen(s) and its susceptibility determine the antimicrobial treatment regimen. However, synovial fluid culture has a limited sensitivity and, hence, does not rule out PJI with sufficient certainty. Reasons for poor sensitivity include low bacterial load in chronic low-grade infections, presence of pathogens adherent on the implant surface as biofilm, prior antimicrobial treatment, delayed transport, or inadequate processing of samples. In contrast, positive culture may represent contamination and does not automatically define infection, except for high-virulent pathogens which are rarely contaminants [5–7].

Few studies assessing a moderate number of episodes revealed an agreement of culture results of preoperative and intraoperative microbiological cultures from 63 to 77% [8–13]. Distinct subgroup analyses considering pathogen virulence or acuity of PJI are lacking. Furthermore, sonication of the retrieved prosthesis was not included in the intraoperative work-up in previous published studies, most likely resulting in an overestimation of the agreement. A misdiagnosis of PJI or the causative pathogen(s) based on the preoperative samples may cause inadequate surgical strategies and insufficient antimicrobial therapy [6, 9].

In this study, we aim to (1) compare the positivity rates of pre- and intraoperative tests, (2) determine the detection rate for individual pathogens, (3) evaluate the concordance of pre- and intraoperative microbiological results, and (4) determine the impact of the antibiotic administration prior to aspiration on their culture positivity. We hypothesized that preoperative microbiological results have limited agreement with intraoperative findings, and hence, initial antimicrobial treatment should not be chosen according to preoperative synovial fluid cultures.

## Patients and methods

### Study population

The study was conducted in a tertiary health-care center with a specialized unit for septic surgery. It was approved by the institutional ethical committee and was performed in accordance with the Declaration of Helsinki. Since 2013, PJI episodes are documented in a prospective cohort as part of the institutional quality-assurance program [14].

### Study design

Patients with culture-positive knee or hip PJI who underwent surgery between February 2011 and August 2018 were screened. Included were episodes, for which samples from both preoperative (synovial fluid) and intraoperative specimens (periprosthetic tissue, synovial, or sonication fluid) were collected and cultured. Preoperative synovial fluid cultures within 6 months before study inclusion were considered. Excluded were episodes with resection arthroplasty and spacer or arthrodesis in place.

Medical information was collected using institutional patient documentation system SAP (SAP NetWeaver SAO GUI for Windows, version 7500.2.9, Walldorf, Germany). Collected data included age and sex of patients, prosthesis location and type (primary or revision), interval between last surgery and arthrocentesis, preoperative diagnostics including white blood cell (WBC) count, serum C-reactive protein (CRP) concentration, synovial fluid leukocyte count and percentage of neutrophils, antimicrobial treatment within 2 weeks prior to joint aspiration or surgery, type of revision surgery, synovial fluid culture (before and/or during surgery), periprosthetic tissue and sonication fluid culture, and histopathological results of periprosthetic tissue.

## Definitions

PJI was confirmed if one or more of the following criteria were present, as previously published [14, 15]: (1) macroscopic visible purulence surrounding the prosthesis, (2) presence of a sinus tract communicating with the joint, (3) increased absolute synovial fluid leukocyte count (> 2000 leukocytes/μl) or percentage of granulocytes (> 70%), (4) significant microbial growth, or (5) positive histopathology, defined as > 23 granulocytes per 10 high-power fields, corresponding to type II or type III periprosthetic membrane [16].

For high-virulent microorganisms, any growth was considered significant, namely ≥ 1 positive tissue or positive synovial fluid culture or sonication culture ≥ 1 colony-forming units (CFU)/ml. In case of low-virulent microorganisms, ≥ 2 positive specimens or > 50 CFU/ml sonication result were required to define growth as significant [17]. Regarding microbiological analysis of synovial fluid, a low-virulent pathogen exclusively detected in pediatric blood culture bottle (i.e., after enrichment) but not in the native vial, was considered non-significant and thereby a contaminant, unless the patient was receiving antimicrobial treatment at time of aspiration.

Detected pathogens were classified according to their virulence into high-virulent (*Staphylococcus aureus, S. lugdunensis, Streptococcus* spp., *Enterococcus* spp., Gram-negative rods, and *Bacteroides fragilis*) and low-virulent (coagulase-negative staphylococci, *Cutibacterium* spp., *Peptostreptococcus*, *Corynebacterium* spp., and *Candida* spp.).

Based on the interval between last (revision) surgery of the prosthetic joint and time of aspiration, all infections were classified as early (≤ 3 months), delayed (3–24 months), or late (> 24 months) [6].

### Diagnostic tests

Synovial fluid was aspirated under sterile conditions preoperatively in the outpatient department or during revision surgery before opening the joint capsule. The aspirate was partitioned into an ethylenediaminetetraacetic acid (EDTA) vial for leukocyte count and differential (1 ml), a native vial (1 ml), and a pediatric blood culture bottle (BacTec PedsPlus/F, Beckton Dickinson and Co., Shannon, County Clare, Ireland) for microbiology. The leukocyte count was determined by flow cytometry using an automated hematology analyzer (XE-2100, Sysmex, Norderstedt, Germany).

For cultures, 0.1 ml of synovial fluid was inoculated on aerobic and anaerobic sheep blood agar plates (BioMérieux, Marcy L’Etoile, France) and incubated 7 days aerobically at 37 °C with 5% CO_2_ and 14 days anaerobically at 37 °C. In addition, 0.5 ml of synovial fluid was inoculated in thioglycolate broth (Beckton Dickinson and Co., Shannon, County Clare, Ireland). The pediatric blood culture bottle was incubated at 37 °C for 14 days or until a positive signal of the instrument was seen.

Periprosthetic tissue samples were homogenized and plated on aerobic and anaerobic blood agar plates, and inoculated in thioglycolate broth, as described for the synovial fluid above. The identification of microorganisms was performed by standard microbiological methods using automated system (VITEK 2, BioMérieux, Marcy L’Etoile, France).

The removed prosthesis was transported to the microbiological laboratory in a sterile air-tight container (Lock&Lock, Frankfurt am Main, Germany) and sonication was performed as previously described [18]. After addition of normal saline covering most of the implant, the container was vortexed for 30 s, sonicated for 1 min at 40 kHz (BactoSonic, Bandelin electronic, Berlin, Germany), and vortexed for 30 s. The resulting sonication fluid was plated in aliquots of 0.1 ml onto aerobic and anaerobic sheep blood agar plates, and 1 ml was inoculated in thioglycolate broth. Cultures were incubated at 37 °C for 14 days and inspected daily for microbial growth. Microorganisms on plates were enumerated as the number of CFU/ml sonication fluid and identified as described above.

For histopathological analysis, periprosthetic tissue samples were fixed in 4% formalin solution and embedded in paraffin, and classified into type I (wear particle induced type), type II (infectious type), type III (combined type), and type IV (indeterminate type) [16].

### Concordance of microbiological results

For evaluation of the concordance of the preoperative compared with the intraoperative microbiological results, categorization was determined as follows: (1) full concordance: identical pathogen detection pre- and intraoperatively (+/+), (2) partial concordance: additional pathogen detection preoperatively (++/+) or additional pathogen detection intraoperatively (+/++), and (3) discordance: pathogen detection only in preoperative samples (+/-) or intraoperative samples (−/+).

### Statistical analysis

Data were recorded using Microsoft^®^ Excel^®^ 2018 (version 16.20; Microsoft, Redmond, USA). Statistical analysis was performed with the software Prism (version 8.0; GraphPad Software, San Diego, USA) and program-package R (version 3.1.3.; https://www.r-statistics.com; Vienna, Austria). Continuous variables were expressed as median or mean values with range, as appropriate. Detection rates were calculated for microbiological and positivity rates for non-microbiological diagnostic methods. Concordance analysis was performed comparing phenotypical test results. For comparison of positivity/detection rates, the Fisher’s exact test was used to estimate *p* values. A *p* value of < 0.05 (two-sided) was defined as statistically significant.

## Results

### Patient demographics and baseline data

Of 610 episodes with hip or knee PJI screened, 167 met the inclusion criteria and were included. The reasons for exclusion were no preoperative arthrocentesis (*n* = 280), no intraoperative sampling (*n* = 24), no pathogen identified in pre- and intraoperative samples (*n* = 126), and no prosthesis in place at time of sampling (*n* = 13). Demographic data and baseline characteristics are presented in Table 1.

**Table 1:** Patient demographic and baseline data of 167 episodes

Variables	All episodes (*n* = 167)
Female sex	99 (59)
Median age, years (IQR)	73 (63–78)
Affected joint	
Hip	76 (46)
Knee	91 (54)
Type of arthroplasty	
Primary prosthesis	73 (44)
Revision prosthesis	94 (56)
Classification according to occurrence of the infection	
Early (< 3 months)	22/164 (14)
Delayed (> 3–24 months)	63/164 (38)
Late (> 24 months)	79/164 (48)
Mean time interval between last surgery and preoperative arthrocentesis, months (range)	45.8 (0.1–284.8)
Patients with antimicrobial treatment	49 (29)
At time of preoperative arthrocentesis	6 (4)
At time of surgery	21 (13)
At time of arthrocentesis and surgery	22 (13)
Type of surgery	
Retention of prosthesis	40 (24)
One- or two-stage exchange of prosthesis	119 (71)
Diagnostic biopsy	8 (5)

### Microbiology data

The proportions of causative microorganisms are shown in Table 2. Polymicrobial infection was detected in 17 episodes (10%). PJI were caused by high-virulent pathogens in 92 (55%) and low-virulent microorganisms in 65 episodes (39%), and 10 episodes were mixed infections with high- and low-virulent pathogens.

**Table 2:** Proportions of isolated pathogens

Pathogen	All episodes (*n* = 167)
Monomicrobial infection	150 (90)
Coagulase-negative staphylococci^a^	55 (37)
*Staphylococcus aureus*	34 (23)
*Streptococcus* spp.^b^	21 (14)
Gram-negative rods^c^	15 (10)
*Enterococcus* spp.^d^	11 (7)
Anaerobes^e^	10 (7)
Other pathogen^f^	4 (3)
Polymicrobial infection	17 (10)

Data are no. (%) of episodes. The percentages were rounded and may not sum 100%

^a^Including *S. epidermidis* (*n* = 43), *S. lugdunensis* (*n* = 6)*, S. hominis* (*n* = 5), and *S. capitis* (*n* = 1)

^b^Including *S. dysgalactiae* (*n* = 9), *S. oralis* (*n* = 5), *S. agalactiae* (*n* = 4), *S. anginosus* (*n* = 1), *S. gallolyticus* (*n* = 1), *S. sanguinis* (*n* = 1), and *S. salivarius* (*n* = 1)

^c^Including *Escherichia coli* (*n* = 8), *Enterobacter cloacae* (*n* = 3), *Campylobacer coli* (*n* = 1), *E. aerogenes* (*n* = 1), *Proteus mirabilis* (*n* = 1), and *Pseudomonas aeruginosa* (*n* = 1)

^d^Including *Enterococcus faecalis* (*n* = 10) and *E. faecium* (*n* = 1)

^e^Including *Cutibacterium acnes* (*n* = 4), *Peptostreptococcus micros* (*n* = 2), *Bacteroides fragilis* (*n* = 1), *Clostridium perfringens* (*n* = 1), *C. avidum* (*n* = 1), and *Parvimonas micra* (*n* = 1)

^f^Including *Candida albicans* (*n* = 2), *C. parapsilosis* (*n* = 1), and *Corynebacterium striatum* (*n* = 1)

### Positivity and detection rates of pre- and intraoperative tests

**Fig. 1:**
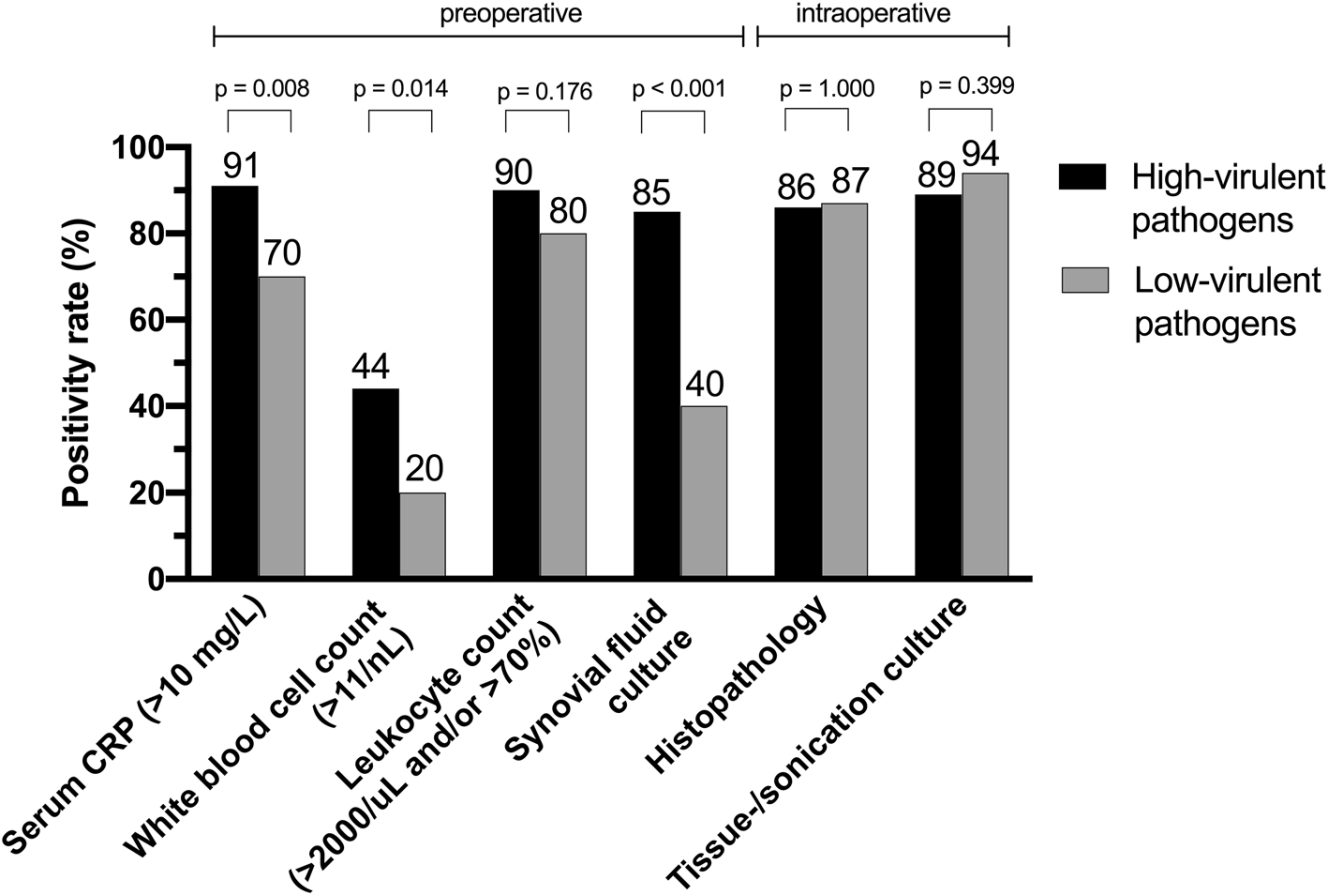
Positivity rate of pre-/intraoperative (non-)microbiological tests according to their virulence: high- (*n* = 92) and low-virulent (*n* = 65). Ten episodes with mixed infections with high- and low-virulent pathogens were excluded

Positivity and detection rates of all preoperative and intraoperative diagnostic tests are shown in Table 3. Preoperative synovial fluid analysis (i.e., increased absolute leukocyte count or elevated percentage of granulocytes and/or positive microbiology) revealed the diagnosis in 137 of 167 (82%) episodes, whereas the intraoperative diagnostic multimodal assessment confirmed PJI in 163 of 167 episodes (98%). Positivity rate of tests in episodes caused by high-virulent and low-virulent pathogens is compared in Fig. 1. A significantly higher positivity rate for serum CRP, WBC count, and preoperative microbiology (synovial fluid culture) was seen in PJI caused by high-virulent pathogens compared to low-virulent pathogens.

**Table 3:** Positivity and detection rates of pre- and intraoperative tests

Diagnostic test	All episodes (*n* = 167)
Preoperative diagnostics serum	
Increased serum CRP (> 10 mg/l)	103/124 (83)
Increased WBC (> 11/nl)	45/124 (36)
Preoperative diagnostics synovial fluid^a^	137/167 (82)
Increased leukocyte count (absolute or percentage granulocytes)	105/125 (84)
Absolute leukocyte count (> 2000/µl)	104/125 (83)
Percentage granulocytes (> 70%)	77/110 (70)
Positive microbiology (synovial fluid)	110/167 (66)
Intraoperative diagnostics	163/167 (98)
Positive histopathology	126/145 (87)
Positive microbiology	153/167 (92)
Synovial fluid	81/138 (59)
Periprosthetic tissue	108/162 (67)
Sonication	95/128 (74)

The results are shown as numbers of episodes (percentages)

^a^Elevated leukocyte count and/or positive microbiology

### Pathogen detection rate

**Fig. 2:**
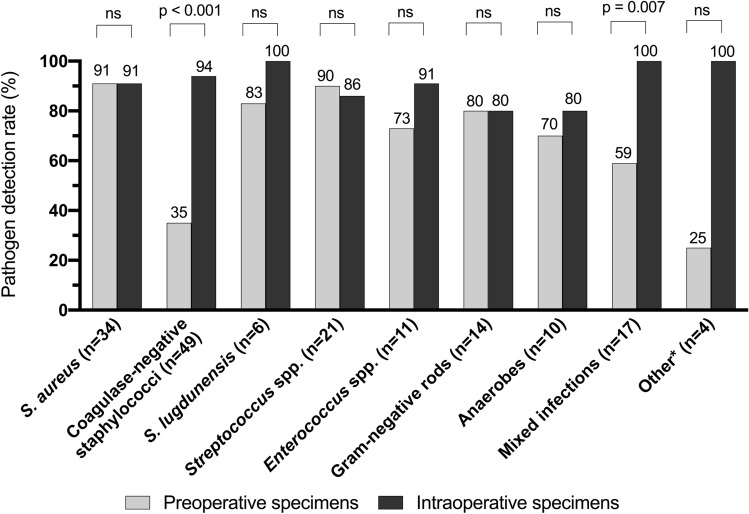
Pathogen detection rates of pre- and intraoperative cultures according to pathogen. *ns* not significant. *Candida albicans (*n* = 2), *Candida parapsilosis* (*n* = 1), and *Corynebacterium striatum* (*n* = 1)

The pathogen was detected in preoperative specimens in 110 of 167 episodes (66%) and in intraoperative specimens in 153 of 167 episodes (92%, *p* < 0.001). The detection rates of pre- and intraoperative specimens for specific pathogens are shown in Fig. 2. Coagulase-negative staphylococci and pathogens of mixed infections were significantly more frequently detected in intraoperative specimen than in preoperatively harvested synovial fluid.

In PJI caused by high-virulent pathogens (*n* = 92), detection rates of pre- and intraoperative cultures were similar (85% vs. 89%, *p* = 0.656), whereas in PJI caused by low-virulent pathogens (*n* = 65), detection rate was significantly lower in preoperative synovial fluid culture than culture of intraoperative specimens (40% vs 94%, *p* < 0.001). Accordingly, in early infections, no significant difference in pathogen detection rate was shown (73% vs 91%, *p* = 0.240). However, detection rate was significantly higher in intra- compared to preoperative analyses in delayed and late infections (63% vs 94%, and 66% vs 91%, respectively, both *p* < 0.001).

The causing pathogen of hip PJI (*n* = 76) was identified by preoperative synovial fluid culture in 46 episodes (61%) and intraoperative specimen culture in 72 episodes (95%; *p* < 0.001). In knee PJI (*n* = 91), preoperative synovial fluid culture revealed the pathogen in 64 episodes (70%) compared to intraoperative specimen cultures in 81 episodes (89%, *p* = 0.003).

### Concordance of pre- and intraoperative microbiological results

**Fig. 3:**
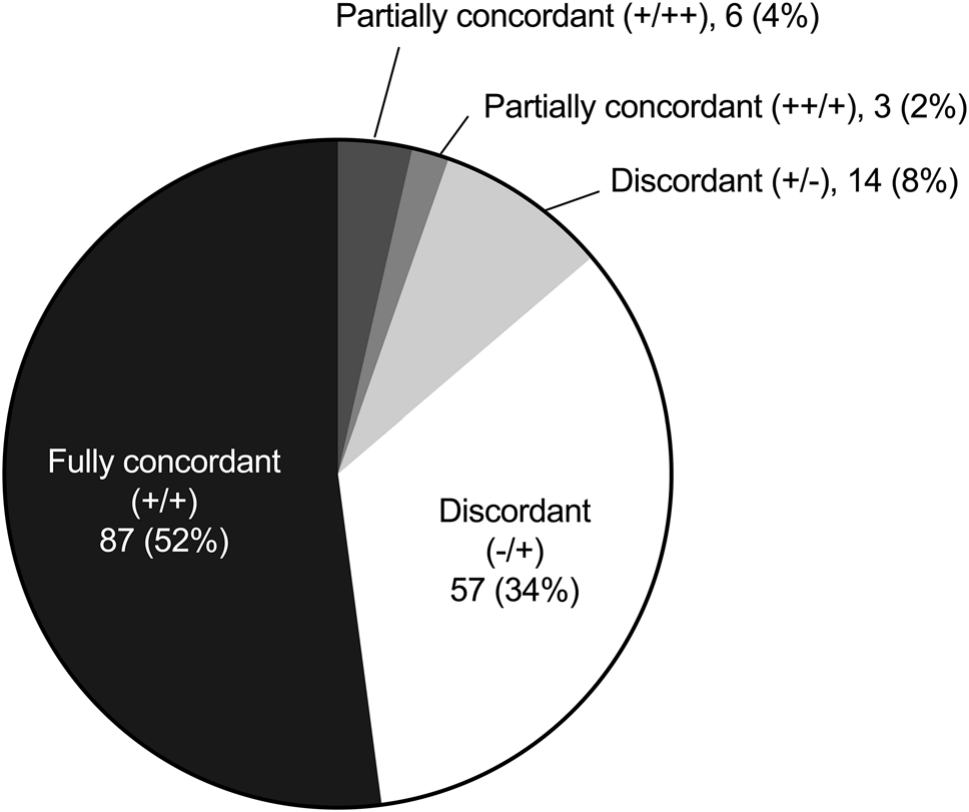
Agreement analysis regarding pathogen detection (pre-/intraoperative culture result). Numbers present absolute number of episodes (percentage)

There was full concordance of pre- and intraoperative microbiological results in 87 episodes (52%). In 80 episodes (48%), discordance or only partial concordance of the microbiological findings was found. Figure 3 summarizes the concordance analysis between pre- and intraoperative microbiological results. The pathogens of concordant and discordant pairs are shown in Table 4. Complete concordance was higher for high-virulent pathogens in comparison to low-virulent pathogens (74% vs 34%, *p* < 0.001). Taking discordant and partially concordant pairs into account, microbiological analysis of synovial fluid harvested by preoperative arthrocentesis missed the causative pathogen or an additional pathogen (significant growth) in 63 episodes (38%).

**Table 4:** Concordance analysis

Diagnostic test		Causative pathogen of the PJI	
Preoperative	Intraoperative		
+	+	87 (52%)	*Staphylococcus aureus* (*n* = 28) Streptococci (*n* = 17) CNS (*n* = 14) Enterococci (*n* = 7) Gram-negative bacilli (*n* = 10) Anaerobes (*n* = 6) *Staphylococcus lugdunensis* (*n* = 5) *Candida albicans* (*n* = 1)
Total full concordance: 87 episodes (52%)			
++ (additional pathogen)	+	3 (2%)	CNS (*n* = 2) *Staphylococcus aureus* (*n* = 2) *Cutibacterium acnes* (*n* = 1) *Escherichia coli* (*n* = 1)
+	++ (additional pathogen)	6 (4%)	Enterococci (*n* = 3) CNS (*n* = 3) Gram-negative bacilli (*n* = 3) *Cutibacterium acnes* (*n* = 2) Streptococci (*n* = 2) *Candida albicans* (*n* = 1)
Total partial concordance: 9 episodes (5%)			
+	−	14 (8%)	CNS (*n* = 3) *Staphylococcus aureus* (*n* = 3) Streptococci (*n* = 3) *Cutibacterium acnes* (*n* = 1) *Enterococcus faecalis* (*n* = 1) Gram-negative bacilli (*n* = 1)
−	+	57 (34%)	CNS (*n* = 36) Enterococci (*n* = 7) Anaerobes (*n* = 5) *Staphylococcus aureus* (*n* = 5) Gram-negative bacilli (*n* = 4) Streptococci (*n* = 3) *Corynebacterium* (*n* = 2) *Candida *spp. (*n* = 2) *Staphylococcus lugdunensis* (*n* = 1)
Total full discordance: 71 episodes (43%)			

*CNS* coagulase-negative staphylococci

### Influence of prior antimicrobial treatment

**Fig. 4:**
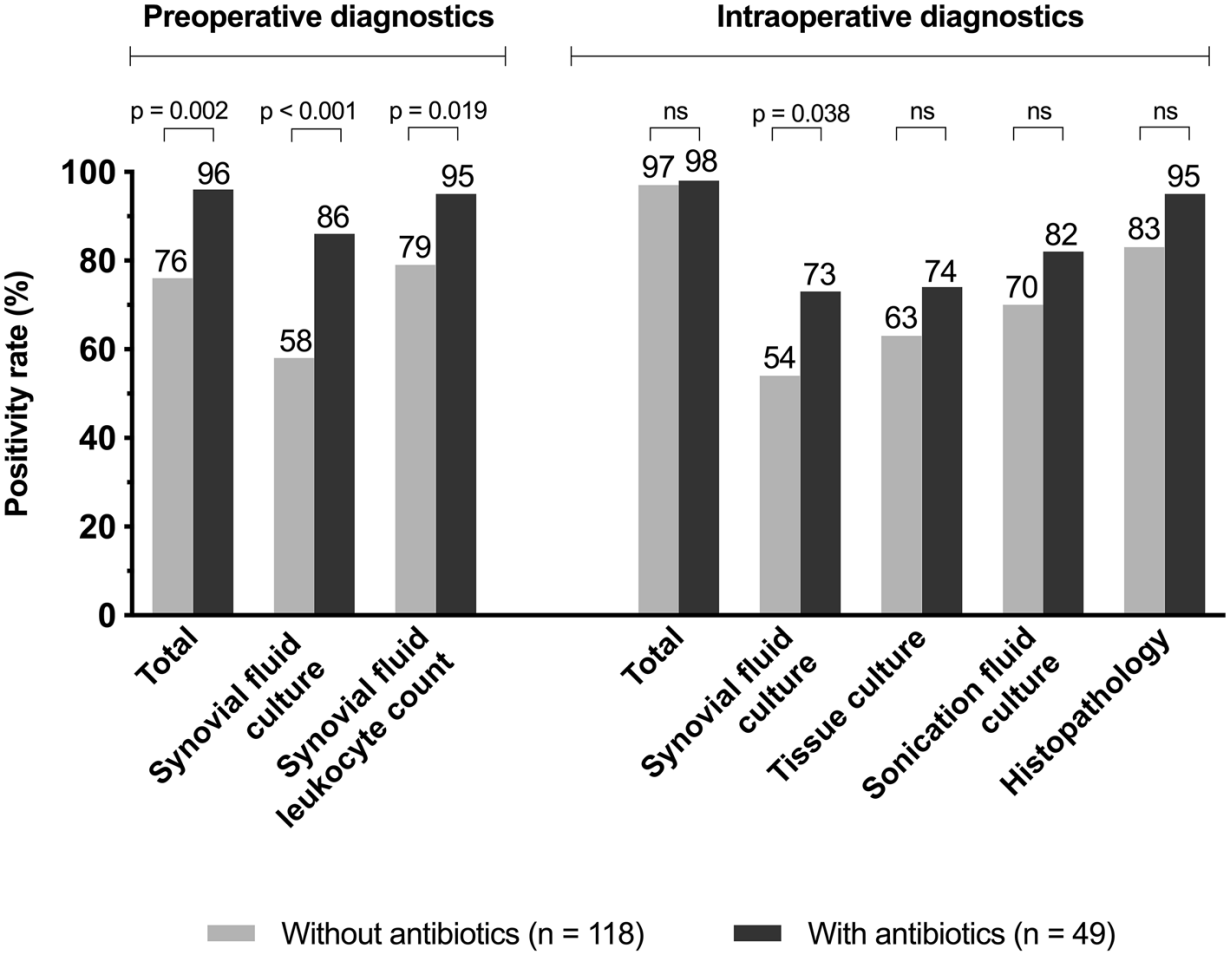
Positivity and detection rate of microbiological and non-microbiological tests in episodes of patients with and without antibiotic pretreatment. *ns* not significant

Antibiotic pretreatment was seen more often in episodes caused by high-virulent pathogens in (34 of 42 episodes [81%], mixed infections excluded), which was a significantly higher percentage compared to the non-pretreated group (54 of 108 episodes [50%], *p* < 0.001). In addition, in the group with antibiotic pretreatment, the CRP was increased (> 10 mg/l) in 39 of 40 episodes (98%, *p* = 0.002). Moreover, all microbiological and non-microbiological tests showed a higher positivity rate in the group with antimicrobial pretreatment compared to the one without antibiotics (see Fig. 4). The pathogen detection rate in preoperative synovial fluid culture was higher in episodes with prior antimicrobial treatment compared to the episodes without antibiotics (42 of 49 [86%] vs 68 of 118 [58%]). Pathogen detection rate of the cumulative intraoperative microbiological methods was similar in both groups (44 of 49 [89%] with prior antibiotics vs. 109 of 118 [92%] without prior antibiotics). In episodes with prior antibiotics, full concordance of pre- and intraoperative results was higher compared to the episodes without pretreatment (35 of 49 [71%] vs. 52 of 118 [44%], *p* = 0.002).

## Discussion

We investigated the diagnostic yield of preoperative cultures of synovial fluid in culture-positive PJI. Aim of this study was in particular to analyze in which situations the preoperative synovial culture was reliably predicting the pathogen and in which conditions the intraoperative results should be awaited before targeting the antimicrobial treatment.

The detection rate of cumulative intraoperative culture results was significantly higher than the culture of synovial fluid sampled by preoperative arthrocentesis. This finding was expected and is explained by the fact, that, in synovial fluid analysis, only planktonic bacteria are collected, while tissue culture and especially sonication reveal their sessile counterparts by detachment of the biofilm [5, 7, 19]. In our study, preoperative synovial fluid cultures revealed the causing pathogen in 66%, which is in line with the previous reports. A meta-analysis of 34 studies assessing the performance of synovial fluid culture reported a pooled sensitivity of 72%. Noteworthy, the included studies used a heterogeneity of definition criteria for PJI and relevant microbiological results and, therefore, the detection of low-grade infections varied widely [20]. The proportion of low-virulent microorganisms largely depends on the applied definition criteria of PJI; the less sensitive the definition criteria are, the lower the detection rate of low-grade infections caused by low-virulent pathogens [21].

The pathogen detection rate was significantly higher in case of infections caused by high-virulent pathogens compared to low-virulent pathogens. Latter typically cause low-grade infections. Similar observations were documented in early (i.e., < 3 months) compared to delayed and late infections (> 3 months), which are typically caused by low-virulent pathogens such as coagulase-negative staphylococci or *Cutibacterium* spp. [7]. These findings contrast the results of previous studies negating influence of the pathogen virulence on its detection rate [10, 22]. Low-virulent pathogens usually cause PJI with subtle clinical signs and symptoms [23–25], as confirmed in our analysis (Fig. 1). The low detection rate of these pathogens in synovial fluid has a high impact on the clinical management of a prosthetic failure, as they are often misdiagnosed as aseptic failures in case of negative microbiology. In our analysis, additional determination of leukocyte count increased the diagnostic yield of preoperative synovial fluid analysis from 66 to 82%. This fact emphasizes the importance of this reliable, rapid, and standardized method in the preoperative setting.

Full concordance was observed only in about half of the episodes (52%). In 38%, the sole or an additional pathogen was missed by preoperative diagnostic arthrocentesis. Four previous studies focused on the agreement on pre- and intraoperative microbiology results [10–13]. In two studies, the *Infectious Diseases Society of America* (IDSA) or *Musculoskeletal Infection Society* (MSIS) criteria were used to diagnose PJI, and in the other two studies, no definition criteria were declared. The concordance of preoperative arthrocentesis in these studies ranged from 63 to 77%, including between 58 and 85 PJI episodes. The higher concordance potentially reflects the higher frequency of high-grade infections, as less “sensitive” PJI definition criteria were applied. For example, using sonication for improved intraoperative pathogen detection, low-virulent pathogens embedded in the biofilm were better identified in the present study, increasing the number of low-grade infections, as previously shown by several authors [18, 26–30]. The poor agreement of pre- and intraoperative pathogen detection should encourage the clinician to select the surgical treatment strategy not only based on synovial fluid culture results, but also consider the patients history (i.e., previous revisions and antimicrobial treatments, which may increase the risk for problematic pathogens).

A minimum of a 2 weeks of antibiotic-free interval is suggested before harvesting microbiological samples to reduce the rate of false-negative results [5, 19, 28, 31]. In our study, the administration of antibiotics did not show any effect on the diagnostic microbiological yield. This result may represent a selection bias, as patients with acute infections more often received prior antibiotic treatment, and in these patients, antimicrobial treatment has little impact on culture positivity predominantly caused by high-virulent pathogens. This observation is corroborated by the fact that positivity rates of all diagnostic tests were higher in the pretreated group, underlining the acuity of the infection.

The main limitation of this study is the retrospective design. Details of medical history were often incomplete, particularly the type and duration of antimicrobial treatment administered before arthrocentesis or surgery. Furthermore, we have not applied the widely used Musculoskeletal Infection Society (MSIS) definition criteria for PJI, hampering the direct comparison of our findings with other publications. In this study, we explicitly used the more sensitive PJI definition criteria including sonication of removed prosthetic components, to particularly improve the detection of slow-growing pathogens such as coagulase-negative staphylococci causing low-grade infection. For these pathogens, the MSIS criteria may not be accurate [1]. However, one of our main conclusions concern exactly this specific subgroup of PJI, which is difficult to diagnose. Finally, including only culture-positive PJI episodes, as performed in other studies, the culture-negative infections would be neglected and distort the real concordance.

In conclusion, preoperative cultures poorly predicted the pathogen of PJI. As clinicians often rely on the preoperative synovial fluid culture to plan the surgical and antimicrobial treatment, this finding is of high relevance. Furthermore, negative synovial fluid culture does not exclude PJI, as approximately one-third of intraoperatively culture-positive episodes were negative in preoperative synovial fluid culture. Rather, a combination of various intraoperative diagnostic methods accurately diagnose PJI and detect the underlying pathogen(s). Finally, broad empiric antimicrobial treatment should be started until intraoperative results are available, irrespective of the pathogen identified in preoperative synovial fluid culture, especially in low-grade infections. In acute infections with clinical and laboratory signs of systemic infection, antimicrobial treatment should be started immediately after arthrocentesis (and collection of blood cultures if hematogenous infection is suspected) and should not be withheld until surgery (as suggested for low-grade infection), as it did not show to decrease the diagnostic yield of intraoperatively taken samples.

## Availability of data and material

Available from the authors by request.
